# Differences in intracranial pressure seen in children and adults could be caused by age differences

**DOI:** 10.1186/2045-8118-12-S1-O21

**Published:** 2015-09-18

**Authors:** Sarah Skovlunde Hornshoej Pedersen, Alexander Lilja Jørgensen, Morten Andresen, Trine Hjorslev Andreasen, Marianne Juhler

**Affiliations:** 1 CSF Copenhagen Studygroup, Denmark

## Introduction

In neurosurgery, one aim of treatment is normalization of intracranial pressure (ICP). Although a range from 7 to 15 mmHg is widely accepted in neurosurgical clinical practice, true reference values are uncertain. No studies compare ICP measurements in children to measurements in adults, and it seems silently assumed that reference values are the same. The same reference range is therefore, regardless of the physiological differences, used for both children and adults.

This study has two aims:

• To clarify if day and night ICP differs between children and adults.

• To examine if age affects ICP.

## Methods

We analysed data from all non-shunted patients undergoing invasive elective diagnostic ICP monitoring from February 2008 to November 2014.

Data from 24 hours of ICP monitoring were separated into day and night sequences. The minimum, maximum, and mean values were determined for both sequences. The first hour of monitoring following probe implantation was discarded to allow for initial stabilization following surgery. To ensure consistency, the same person analysed all ICP monitoring sessions.

## Results

We included 130 patients (58 children, mean age=9.0 years, range 1-17. 72 adults, mean age=50.1 years, range 18-85).

We found that nocturnal ICP increased in 95% of the patients. Looking at ∆ICP (for day and night pressure) the intrapersonal difference was nearly identical in children (mean 6.6 mmHg) and adults (6.3 mmHg) p=0.798. However, there were noteworthy differences between children and adults;

• Children had both higher mean ICPday (6.3 vs. 2.5 mmHg, p<0.001) and mean ICPnight (12.6 vs 8.9 mmHg, p<0.001) than adults.

• In adults we found that both ICPday and ICPnight decreased with age (decrement of 0.1 mmHg per year).

• In children only ICPday decreased with age (decrement of 0.45 mmHg per year).

## Conclusion

We found that both children (p<0.001) and adults (p<0.001) displayed higher ICP values at night, and the difference between night and day was comparable between the groups.

Measured ICP values were higher in children than in adults both day and night.

We furthermore found that ICP could be described as a linearly decreasing function of age. This could indicate that the ICP reference values for children should be different than those for adults.

The results are important for clinical management of paediatric ICP and contribute to the understanding of the basic physiology of the brain.

